# Testing the credibility, feasibility and acceptability of an optimised behavioural intervention (OBI) for avoidant chronic low back pain patients: protocol for a randomised feasibility study

**DOI:** 10.1186/1745-6215-14-172

**Published:** 2013-06-13

**Authors:** Tamar Pincus, Shamaila Anwar, Lance McCracken, Alison McGregor, Liz Graham, Michelle Collinson, Amanda J Farrin

**Affiliations:** 1 Department of Psychology, University of London, Royal Holloway, Egham, Surrey TW20 0EX, UK; 2 Clinical Trials Research Unit, University of Leeds, Leeds, LS2 9JT, UK; 3 Health Psychology Section, Psychology Department, King’s College, 5th Floor Bermondsey Wing Guy’s Campus, London SE1 9RT, UK; 4 Department of Surgery and Cancer, Faculty of Medicine, Imperial College London, Charing Cross Hospital, London W6 8RF, UK

**Keywords:** Low back pain, Psychology, Contextual Cognitive Behavioural Therapy, Fear avoidance

## Abstract

**Background:**

Chronic back pain continues to be a costly and prevalent condition. The latest NICE guidelines issued in 2009 state that for patients with persistent back pain (of between six weeks and twelve months duration), who are highly distressed and/or disabled and for whom exercise, manual therapy and acupuncture has not been beneficial, the evidence supports a combination of around 100 hours of combined physical and psychological treatment. This is costly, and may prove unacceptable to many patients. A key recommendation of these guidelines was for further randomised controlled trials (RCTs) of psychological treatment and to target treatment to specific sub-groups of patients. Recent trials that have included psychological interventions have shown only moderate improvement at best, and results are not maintained long term. There is therefore a need to test theoretically driven interventions that focus on specific high-risk sub-groups, in which the intervention is delivered at full integrity against a credible control.

**Methods/design:**

A feasibility study of a pragmatic randomised controlled trial comparing psychologist-delivered Contextual Cognitive Behavioural Therapy (CCBT) against Treatment As Usual (TAU) physiotherapy delivered by physiotherapists for the treatment of chronic lower back pain in ‘avoidant’ patients. Ninety-two patients referred for physiotherapy will be recruited and randomised on a 1:1 basis to receive CCBT or TAU. Treatment groups will be balanced by centre and pain interference score. Primary outcomes include assessing the credibility and acceptability of the intervention, and to demonstrate proof of principle through a greater change in pain acceptance in the CCBT arm, measured by the Acceptance and Action –II and the Chronic Pain Acceptance questionnaires. In addition, the feasibility of carrying out a full trial will be explored with reference to recruitment and follow-up rates including the assessment of the burden of outcome measure completion. Secondary patient outcomes include disability, pain, fear of movement, mood, quality of life, and global recovery. Outcomes are measured at three and six months post-randomisation.

**Discussion:**

This paper details the rationale, design, therapist training system and recruitment methods to be used in a feasibility study which will inform the design and efficient implementation of a future definitive RCT.

**Trial registration:**

ISRCTN43733490

## Background

Chronic musculoskeletal pain, including the primary complaint of back pain, remains a costly and prevalent problem. Back pain has been identified as a leading cause of disability world-wide, with an estimated 632 million people affected [1]. During the course of one year 20% of UK adults consult their General Practitioner (GP) about a musculoskeletal problem, making this the second most frequent reason for consultation after respiratory disease. The most common musculoskeletal presentation is spinal (low back and neck) pain, with 38% of adults affected in any one year at an estimated cost to the National Health Service (NHS) of £1 billion per annum [2].

There is an evidence-based consensus that certain psychological factors (conceptualised as yellow flags [3]), form obstacles to recovery, and should, therefore, be targeted by interventions. In the UK, the National Institute for Health and Clinical Excellence (NICE) guidelines (2009) for the management of persistent low back pain with a duration of six weeks to twelve months recommend a combination of physical and psychological treatment for a sub-group of patients who are high in disability and distress [4]. The evidence suggests that around 100 hours of treatment over 8 weeks are beneficial, but this is costly, and may prove unacceptable to many patients. A key recommendation of these guidelines was for further randomised controlled trials (RCTs) of psychological treatment. To date, the evidence from trials of biological, psychological and social interventions (and combinations of these) has been disappointing, indicating at best small to moderate improvements in outcomes when compared with treatment as usual. In addition, these effects are often not maintained at long-term follow up [5].

Systematic reviews of trials that have tested psychological interventions for chronic pain [6] report considerable gaps in the evidence, including failure to examine long-term effects; failure to select appropriate risk-groups that are most likely to benefit from the intervention; insufficient methodological quality; and little evidence to compare directly the efficacy of different interventions. Reviews recommend that future intervention should be more focused and theory-driven. A consensus paper attempting to clarify the disappointing results from psychological interventions has emphasised the need for better integrity in treatment delivery, and selection of at-risk patients [7]. A recent trial has demonstrated that stratified care is a promising approach: patients in the sub-groups for targeted treatment for back pain screening (STarT Back) trial were screened for low, medium and high risk, the latter including psychological factors such as catastrophic thinking and low mood. The high-risk group received CBT-informed physiotherapy. Overall, the trial demonstrated that screening and matching treatment for specific risk groups improved outcomes significantly more than physiotherapy alone [8]. However, questions remain about the optimal intervention for the high risk group, especially in reference to long-term outcomes.

We thus aimed to follow the recommendations for improving trials of psychological treatment for high-risk patients, by developing and testing an intervention that is: theoretically driven; explicitly links the intervention to a hypothesised risk factor in patients; where hypothesised outcome can be measured reliably; delivered to a quality that reflects the integrity of the intervention in reference to training and content; methodologically implemented to allow for adequate power to test the effectiveness of the intervention; implementable in UK-based National Health Service NHS settings; and is acceptable to patients.

We have identified a promising approach for treating a sub-section of the high-risk group of patients. Contextual Cognitive Behavioural Therapy (CCBT) is an approach within the wider family of cognitive and behavioural therapies, based on the treatment model of Acceptance and Commitment Therapy (ACT) [9] and including methods of mindfulness [10,11]. The intervention aims to increase psychological flexibility and acceptance, thus impacting on function and improving quality of life rather than reducing pain. There is some evidence to suggest that the intervention is promising, but there are no conclusive high- quality RCTs carried out on at-risk (avoidant) patients in pain populations. In addition, the level of acceptability to patients has not been assessed.

Although the intervention may be suitable for a variety of high-risk groups, we elected to focus on a specific, evidence-based risk factor, namely, avoidance. A fundamental aspect of both distress and disability is withdrawal from and reduced participation in daily activity, social contact and work. This avoidance process is considered a key obstacle to recovery, and is described in the Fear Avoidance Model (FAM) [12]. CCBT conceptualises fear avoidance processes in a wider form than the standard FAM and changes the focus of traditional pain management. In CCBT any psychological experiences that coordinate avoidance can become the focus in treatment with the emphasis on the influences exerted by these experiences and not solely on changing their content or frequency. CCBT includes both traditional methods for changing behaviour, and newer methods developed from the theory underpinning ACT and mindfulness. These newer methods are predominantly experientially based, and address such processes as acceptance and patient values [9,13-15]. CCBT also includes a specific approach to the therapeutic relations designed to enhance treatment impact. The primary defining feature of CCBT is that it is focused on enhancing patient psychological flexibility.

A definitive RCT is planned for the future, however, to inform such a large trial the feasibility study described below is being conducted.

### Aims and objectives

The aim of this study is to test the acceptability and credibility of CCBT against best-practice physiotherapy (treatment as usual (TAU)) for people with chronic back pain with associated avoidance of daily activities. In addition, we aim to test the feasibility of implementing a full scale RCT.

The primary objectives are:

•To determine whether CCBT is an acceptable and credible intervention for NHS patients with persistent back pain and avoidance in comparison to TAU

•To assess recruitment processes and study uptake to inform the feasibility of a definitive RCT

•To assess follow up response rates and level of missing data

•To assess the burden of measurement tool completion

•To demonstrate proof of principle by gathering information about the process of change between the two treatment arms (that is, whether the effect of CCBT in enhancing acceptance post-intervention is sufficiently promising compared to the control to warrant a full trial)

The secondary objectives are to measure long-term changes in quality of life outcomes relating to mood, pain, disability and functioning.

## Methods/design

### Trial design

OBI is a pragmatic multi-centre randomised controlled feasibility study that has been designed to assess the methodology proposed for use in the definitive RCT.

### Settings and participants

A total of 92 participants will be recruited from four NHS musculoskeletal/physiotherapy services who have agreed to take part in this study. Patients meeting the following criteria are eligible for study entry:

•Over 18 years of age

•Suffering from chronic lower back pain of at least three months duration

•Suitable for physiotherapy-led treatment

•Not requiring referral to any other department/service

•Classified as avoidant (defined as endorsing any of the three psycho-social questions from the high-risk sub-group on the Sub-groups for Targeted Treatment for Back Pain Screening Tool (STarT Back) [16] and a score of 38 or more on the Tampa Scale for Kinesiophobia (TSK) [17]

Patients meeting any of the following criteria will not be eligible for study entry:

•Currently receiving psychological treatment

•Presence of sciatica (burning throbbing and radiating leg pain of spinal origin confirmed by performing the straight-leg raise test)

•Presence of a progressive disorder (for example, chronic obstructive pulmonary disease (COPD), cardiovascular disease, rheumatoid arthritis, lupus, ankylosing spondylitis and neoplastic disease)

•Pregnancy

•Insufficient proficiency in English to comply with treatment or provide data

•Involved in ongoing litigation relating to the pain condition

Determining eligibility for participation will be a multi-stage process; some of the eligibility criteria are established upfront during review of the referral by the on-duty physiotherapist (for example, age, duration of symptoms), others during review of the completed screening questionnaire, telephone contact with the patient or during the physiotherapy assessment. Rates of referrals, eligibility, consent, and randomisation are monitored at each stage to assess trial uptake.

### Participant identification and recruitment process

**Figure 1:**
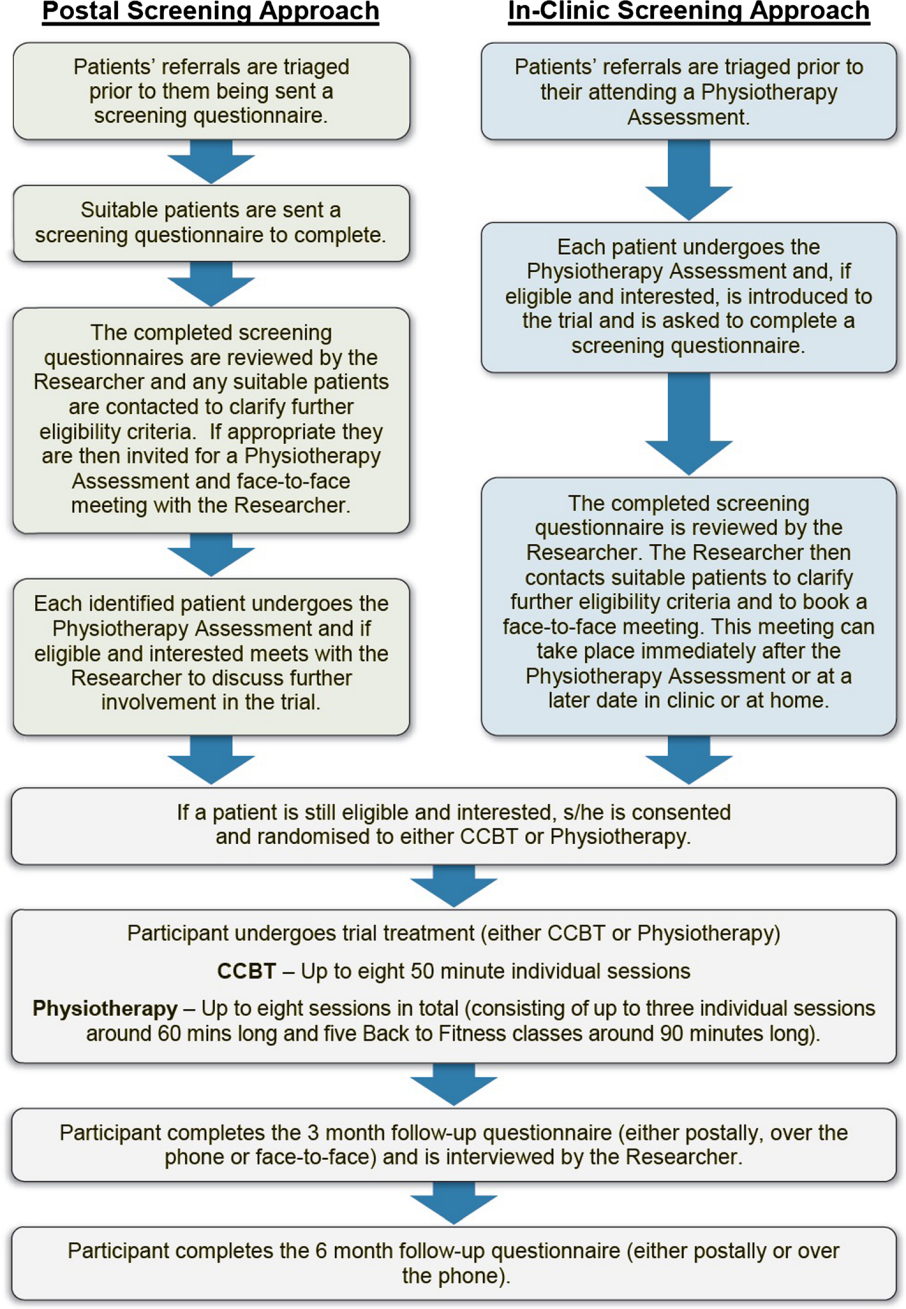
Participant identification, recruitment and follow-up procedure.

The participant identification, recruitment and follow up procedure is shown in Figure 1. In summary, all referrals made to the musculoskeletal/physiotherapy services involved in the study will be triaged. Those patients suitable will be asked to complete a screening questionnaire and consent to be contacted by the researcher. Questionnaires will be sent out by post or given in-clinic. The method used will be dependent on a centre's usual triage and assessment procedures and the time lag between referral and first appointment. If the responses provided by the participant indicate that they are eligible, and if they consent to being contacted, the researcher will contact patients to further clarify eligibility. If eligible, the patient is invited to attend a physiotherapy assessment and a face-to-face meeting with the researcher. The physiotherapy assessment will ensure that the participant is not suffering from sciatica or any other progressive disorders that may require a different clinical pathway. Eligible patients will then meet face-to-face with the researcher to learn more about the study, what participation involves, ask any questions they may have, and provide written informed consent. Baseline data will be collected (as detailed in the data collection section below) prior to randomisation.

It is made clear in all information provided to participants that they do not have to complete the questionnaire if they do not wish to, nor do they have to participate in the trial. The patient can withdraw from the study at any time and without prejudicing any further treatment.

### Treatment

#### TAU – physiotherapy

A pragmatic approach of a class intervention in the form of a Back-to-Fitness class has been implemented. Such classes are commonplace in physiotherapy departments across the UK. They are supported by the academic literature, and permit a degree of standardisation for the purposes of an RCT.

Classes typically comprise five sessions each lasting up to 90 minutes, with a maximum of ten patients per class spread out over a period of five weeks. Classes include study participants and non-study participants. As classes run several times a week, participants who miss a session have the opportunity to re-schedule. Classes will be delivered by a senior physiotherapist, and the timing and location of the classes will be in line with each department's routine procedures. The basic premise is that exercise rather than a pain management intervention should form at least 60% of each class. Classes will include components such as: general fitness training, patient discussion, relaxation, goal setting, core stability, stretching and exercises, pacing, self-management and avoidance of recurrence. Participants will also be given tips of the day and handouts. Classes can include educational elements such as discussions of fear avoidance, but explicit use of CCBT or other psychological methods designed to target fear and avoidance, such as systematic exposure, or methods focused on enhancing psychological flexibility are to be avoided.

Prior to attending the Back-to-Fitness classes, participants may also require individual physiotherapy sessions to prepare them to receive group therapy. The aim of these individual sessions will be to tackle any issues that may prevent patients from adhering to treatment, thus minimising the chances of patients dropping out. The number of individual physiotherapy sessions offered to patients will be at the discretion of the physiotherapist and will be dependent on issues such as severity of pain and avoidance characteristics, but should not exceed more than three individual sessions, each no more than 60 minutes in duration.

#### Intervention arm – CCBT

CCBT is an approach based on a functional contextual theory of human behaviour and a general approach to behavioural treatment called ACT. It seeks to create behaviour change by processes of identifying occasions where behaviour patterns exhibit a quality of psychological inflexibility, and intervening with these occasions to promote psychological flexibility. Psychological flexibility is the capacity to persist with a behaviour pattern or change it to effectively reach goals in a way that appreciates what situations afford. One of the unique features in applying psychological flexibility is that it addresses dysfunctional cognitive influence not by suggesting a change in the content of thoughts but by seeking to reduce the exclusive dominance of cognitive influence and by bringing behaviour more under the influence of direct experience.

Participants randomised to CCBT will receive a maximum of eight sessions (each lasting around 50 minutes) with a Health and Care Professions Council-registered practitioner psychologist trained in the use of CCBT methods for treating patients with chronic pain. The first session will include initial work on demonstrating an understanding of the patient's problems, building rapport and providing a basic description and rationale for the treatment approach. Subsequent sessions will focus on the use of experiential, exposure-based, and mindfulness-based methods to enhance acceptance, a present-moment focus, awareness, and values-based action. The number of subsequent sessions will be determined by agreement between the patient and therapist that patient's goals have been met. Goals-based ‘committed actions’ will be promoted regularly, both in session and between sessions, and built into integrated, generalised, and long-term patterns of behaviour.

Therapists delivering CCBT are trained to competence with an intensive two-day experiential workshop, completion of a currently available therapist training workbook under supervision, and supervision of a series of at least three individual patients with chronic lower back pain by an expert in CCBT. The number of training cases completed depends on how competent the therapist is in delivering CCBT (each therapist can complete up to five training cases). A core competency rating form will be used in training to track therapists’ level of developing skill for the delivery of CCBT [18]. To maintain faithful treatment delivery throughout the study, supervision will be provided on a two-weekly basis with extra supervision available for the first three patients recruited at each centre to ensure treatment is being delivered optimally as per the CCBT treatment manual. Supervision includes regular review of audio-taped CCBT sessions.

### Randomisation

Participants will be randomised on a 1:1 basis to receive either CCBT or TAU and will be stratified by centre and pain interference score [19-21]. Randomisation will occur through the Clinical Trials Research Unit (CTRU) 24-hour automated randomisation system.

### Randomisation of training cases

Participants will be randomised as training cases until the CCBT trainer deems the therapist competent in the delivery of CCBT. Randomisation of training cases is necessary to ensure therapists have a pool of participants on whom to practice CCBT treatment methods.

### Blinding

Participant and clinician blinding is not possible due to the nature of the intervention; nor is researcher blinding, as the researcher conducts a semi-structured interview at three months, which includes treatment-specific topics and questions.

### Primary and secondary outcomes

The primary outcomes of interest include credibility and acceptability of CCBT in the treatment of back pain, and the feasibility of recruitment and completion of outcome measures to the trial. These will be assessed as follows:

•Credibility of treatment to patients

∘ First two modified (prospective) questions of the Borkovec and Nau questionnaire prior to treatment (expectation) [22]

∘ Original Borkovec and Nau questionnaire at three months (satisfaction) [22]

∘ Patient interview at three months: a qualitative semi-structured interview with the researcher.

•Acceptability of treatment to patients

∘ Number of sessions attended

∘ Reasons for early withdrawal from treatment

∘ Patient interviews at three months

•Acceptability and credibility of the treatment to therapists

∘ Interview at end of study

•Feasibility of recruitment process

∘ Number eligible for screening questionnaires

∘ Number of screening questionnaires completed and returned

∘ Numbers attending interview with researcher

∘ Numbers randomised entered into the trial

∘ Reasons for non-participation and ineligibility

∘ A comparison of the postal and in-clinic screening and recruitment methods

•Feasibility of assessment tools

∘ Time taken to complete questionnaires at all time points

∘ Number of missing items

∘ Follow up rates

∘ Patient interviews at three months - acceptability of measurement completion

•Proof of principle (process of change due to treatment)

∘ Chronic Pain Acceptance Questionnaire (CPAQ) [23] at three months

∘ Acceptance and Action Questionnaire-II (AAQ-II) [24] at three months

The secondary outcomes (measured at three and six months) include:

•Disability, measured by the Roland Morris Disability Questionnaire (RDQ) [25].

•Pain, measured by Brief Pain Inventory (BPI) [18-20]. Although CCBT does not aim to reduce pain, a measurement of pain is needed to test the randomisation process and be included as a covariate in the final analysis.

•Fear of movement, measured by the TSK [17]. The TSK measures the extent in which patients with chronic pain experience fear of movement or injury/re-injury.

•Mood measured using the Hospital Anxiety and Depression Scale (HADS) [26]

•Quality of life measured by the Euroquol 5D (EQ-5D™) [27,28] and the Short-Form 12 (SF-12V2™) [29].

•Recovery, measured by the Modified Patient Global Impression of Change (MPGIC) [30,31].

The health economics section of this feasibility study will assess the acceptability of obtaining resource use data from patients about both personal and NHS costs (a societal perspective). A *de novo* questionnaire based on existing questions used in other back pain studies has been developed. This questionnaire also includes a short selection of questions to test the representativeness of the patient population against those reporting musculoskeletal problems in the British Household Panel Survey (BHPS).

### Data collection

Table 1 details the information collected at the different time points. Three- and six-month questionnaires will be posted to the participants by the CTRU. The three-month questionnaire includes a £10 voucher as an unconditional incentive. At the same time the CTRU will prompt the researcher to remind the participant that they will receive a questionnaire to complete, and ideally this should be completed and returned before they meet for the three-month interview. The CTRU will inform the researcher if a questionnaire has not been returned within two weeks, in which case the questionnaire will be completed in the face-to-face interview. If the participant does not wish to be interviewed face-to-face and has not completed the questionnaire the researcher will attempt to complete the questionnaire with the participant over the telephone. Telephone completion will also be offered to non-responding participants at the six-month follow up.

**Table 1:** Time points at which measures and data are collected

	**Screening**^**a**^	**Baseline**	**During treatment**	**Three month**	**Six month**
Gender	X*				
Age	X*				
Source of referral	X*				
Date of referral	X*				
Suitability for OBI (from information provided on referral)	X*				
Has the patient been sent a questionnaire?	X*				
BPI	X	X		X	X
	(pain location only)	(full)		(full)	(full)
TSK	X			X	X
STarT Back	X				
Demographic information (age, gender, ethnicity)		X			
History of the pain condition		X			
Contact details		X			
CPAQ		X		X	X
AAQ-II		X		X	X
RDQ		X		X	X
SF-12		X		X	X
EQ-5D		X		X	X
HADS		X		X	X
Expectation and satisfaction with treatment		X		X	
		(expectation)		(satisfaction)	
Date of each scheduled CCBT or physiotherapy session and whether the session was attended			X		
Reasons for non-attendance or early withdrawal			X		
Details of any concomitant treatments			X		
Details of any treatments the participant has been referred on to			X		
Recovery (MPGIC)		X		X	X
Health economics		X		X	X
Help questions (How long it has taken to complete the questionnaire? Did anyone help you to complete the questionnaire?)	X	X		X	X
Face-to-face interview		X		X	

^a^ An asterisk indicates that the data is collected for all patients triaged.

AAQ-II: Acceptance and Action Questionnaire II; BPI: Brief Pain Inventory; CCBT: Contextual Cognitive Behavioural Therapy; CPAQ: Chronic Pain Acceptance Questionnaire; EQ-5D: Euroquol 5D; HADS: Hospital Anxiety and Depression Scale; MPGIC: Modified Patient Global Impression of Change; OBI: Optimised Behavioural Intervention; RDQ: Roland Morris Disability Questionnaire; SF-12: Short Form 12; STarT Back: Sub-groups for Targeted Treatment for Back Pain Screening; TSK: Tampa Scale for Kinesiophobia.

The three-month interview schedule ascertains participant’s views on the acceptability and credibility of treatment and the burden of questionnaire completion.

### Monitoring treatment attendance and adherence

#### Treatment attendance

For both treatment arms, attendance at treatment sessions is monitored via completion of a treatment attendance form following all scheduled treatment sessions. This form records the participants’ attendance and reasons for non-attendance and participant withdrawal from treatment (either by the therapist or participant themselves), concomitant treatments and whether the participant has been referred on for other treatment.

#### Treatment adherence and integrity

In addition to the treatment attendance forms, for each treatment session attended, a session rating form is also completed by the treating physiotherapist or psychologist. These forms have been designed to ascertain which components of the treatments were covered and are specific to the treatment arm. In the CCBT arm, all sessions will also be audio-taped and a sample of these coded by an independent reviewer, using an agreed rating scale developed by the CCBT expert. To ensure treatment adherence in the TAU arm, a sample of the physiotherapy classes will be observed by the physiotherapy expert and the components covered recorded.

### Sample size

We plan to recruit 92 participants in total, randomised equally to the TAU and CCBT arms. As this is a feasibility study, formal power calculations are not appropriate, as the study is not designed to test for a difference between treatments. Recommendations for feasibility studies propose that the analysis dataset comprises a minimum of 30 participants for each arm in order to estimate parameters for future sample size calculations [32-34]. We anticipate that the combined total of non-compliance and loss to follow up will be no more than 35% (that is, 25% due to loss to follow up and 10% due to non-compliance) and therefore, aim to recruit 46 patients in each arm, to account for potential missing data. Part of the rationale for a feasibility study is to gather data to inform the design of a definitive study; hence, estimates for non-compliance and loss to follow up rates in this patient group will be generated by the end of the study and will feed into power calculations for the full trial.

### Planned analyses

The analyses will be descriptive in nature and provide estimates of key trial parameters for the definitive RCT. A combination of qualitative and quantitative measures will be used to address the research questions relating to the acceptability and credibility of CCBT and the feasibility of conducting a definitive trial in the future. Statistical analyses will be on an intention-to-treat basis with participants being analysed according to their randomisation allocation. Data will be analysed at the end of the study when all data collection, entry and validation is completed. There are no planned sub-group analyses. As this is a feasibility study, analysis will focus on confidence interval estimation, rather than hypothesis testing. Credibility data (expectation and satisfaction) will be summarised by group and centre, together with 95% confidence intervals. The number of participants missing sessions, and withdrawal (from treatment, follow up or both) in each arm will be reported overall, by centre and by therapist.

The success of recruitment strategies will be measured by summarising eligibility, consent and randomisation rates, both overall and by centre. The burden of measurement tool completion will be assessed by summarising follow up rates and the time taken at baseline and follow up for completion of the measurements. The level of missing data (for individual items and for entire outcome measures) will also inform the assessment of measurement tool acceptability.

Qualitative analyses will be carried out independently by two researchers on transcripts from the interviews conducted with consenting patients in each treatment condition. We will use directed content analysis [35] to explore consensus, and present the analysis backed by verbatim quotes to illustrate main points. Errors, omissions and commission will be discussed and resolved between the researchers.

To investigate the process of change and to generate evidence of proof of principle, we will report mean change from baseline in the three-month CPAQ scores separately for the randomised groups with their 95% confidence intervals. CCBT is expected to show a greater change in the CPAQ than TAU. In addition, we use exploratory plots and measures of correlations to investigate the relationship between levels of change in acceptance and disability at follow up.

Secondary outcome measures relating to quality of life, mood, pain and global recovery post-treatment at six months will be summarised by point estimates, variability estimates and 95% confidence intervals presented by randomised group at each time point.

### Ethics and RD approval

A favorable ethical opinion was granted for this study by the West London Research Ethics Committee (REF: 11/H0706/9). Research governance approval for all participating sites was gained via the Coordinated System for gaining NHS Permission. All information collected during the course of the study will be kept confidential. Information will be transferred, held securely on paper and electronically, and archived in compliance with the 1998 Data Protection Act at the CTRU, Royal Holloway University of London and at participating centres.

### Trial governance

The Trial Management Group (TMG), comprising the chief investigator, the CTRU team and co-investigators, provides overall management of the study including clinical set-up and training, centre set-up in preparation for recruitment, promotion of the study and interpretation of the results.

An independent Trial Steering Committee (TSC) provides independent and scientific oversight of the study on behalf of the sponsor (Royal Holloway, University of London) and the funder (Arthritis Research UK). The TSC is composed of an independent chair and four other independent members (including one lay member) whose role it is to ensure the study is conducted in accordance with good clinical practice.

## Discussion

The OBI study opened to recruitment in August 2011 and addresses an important gap in the evidence on how to provide effective interventions for people with chronic lower back pain, who are at high psychosocial risk. The main issues encountered in the set-up and implementation have revolved around the training of psychologists to deliver CCBT, the development of effective recruitment processes that fit across all sites (which vary significantly in their structure and function) and the development of successful strategies to procure three- and six-month follow up data from this difficult high-risk patient population. Despite these issues the trial is recruiting well and invaluable information has been gathered about the design and logistical issues that will inform the design, set-up and implementation of a definitive RCT. Feasibility studies, therefore, play an invaluable part in health research. Methodological rigor is achieved through a well-thought-out and conducted study with clear aims and objectives, which result in higher quality, well-designed definitive RCTs.

## Abbreviations

AAQ-II: Acceptance and Action Questionnaire-II; AR-UK: Arthritis Research UK; BPI: Brief Pain Inventory; CCBT: Contextual Cognitive Behavioural Therapy; CLRN: Comprehensive Local Research Network; CPAQ: Chronic Pain Acceptance Questionnaire; CTRU: Clinical Trials Research Unit, Leeds; EQ-5D: Euroquol 5D; GP: General Practitioner; HADS: Hospital Anxiety and Depression Scale; NHS: National Health Service; MPGIC: Modified Patient Global Impression of Change; NICE: National Institute for Health and Clinical Excellence; OBI: Optimised Behavioural Intervention; RCT: Randomised Controlled Trial; RDQ: Roland Morris Disability Questionnaire; SF-12: Short-Form 12; STarT Back: Sub-groups for Targeted Treatment for Back Pain Screening; TAU: Treatment As Usual; TMG: Trial Management Group; TSC: Trial Steering Committee; TSK: Tampa Scale for Kinesiophobia.

## Competing interests

The authors declare they have no competing interests.

## Authors’ contributions

The authors provided the following contributions to this paper: TP, the conception and design of the study, acquisition and interpretation of data and drafting of this paper; LM, the development of CCBT, the training of psychologists in CCBT, the design of the study and commenting on the draft of this paper; AM, the standardisation of the physiotherapy classes, the design of the study and commenting on the draft of this paper. SA; the set-up, implementation and day-to-day management of the study and drafting of the paper; MC, the development of the statistical analysis plan and commenting on the draft of this paper; LG, the design of the study and commenting on the draft of this paper; AJF, the conception and design of the study, statistical guarantor and acquisition, and drafting of this paper. All authors read and approved the final manuscript.

## Authors’ information

Professor Tamar Pincus holds a Masters degrees in experimental research methods in psychology from University College London (UCL), and epidemiology, from Cambridge University, as well as a PhD from UCL. Her research has embraced a variety of methodologies, including experimental, epidemiological and qualitative. Her research has included investigation of attention and recall in pain patients; the psychological predictors for poor outcome in low back pain, and the study of clinicians’ beliefs and attitudes in low back pain. Recently the focus of her research has moved to investigating the effectiveness of interventions through randomised controlled trials, and throughout she has collaborated closely with researchers from many disciplines, including doctors, physiotherapists, osteopaths, chiropractors and clinical psychologists, from a multitude of institutions. She has also convened the international consensus group to establish what factors and measures should be included in prospective cohorts investigating the transition from early to persistent back pain.
